# Contaminants of emerging concern in the Maumee River and their effects on freshwater mussel physiology

**DOI:** 10.1371/journal.pone.0280382

**Published:** 2023-02-01

**Authors:** Ieva Roznere, Viktoriya An, Timothy Robinson, Jo Ann Banda, G. Thomas Watters

**Affiliations:** 1 Department of Evolution, Ecology, and Organismal Biology, The Ohio State University, Columbus, Ohio, United States of America; 2 Faculty of Biology, University of Latvia, Riga, Latvia; 3 Department of Mathematics and Statistics, University of Wyoming, Laramie, Wyoming, United States of America; 4 U.S. Fish and Wildlife Service, Gloucester, Virginia, United States of America; Jiangsu University, CHINA

## Abstract

Contaminants of emerging concern pose a serious hazard to aquatic wildlife, especially freshwater mussels. The growing number of contaminants in aquatic systems requires scientists and managers to prioritize contaminants that are most likely to elicit a biological response for further monitoring and toxicological testing. The objectives of this study were to identify a sub-category of contaminants most likely to affect *Pyganodon grandis* and to describe alterations in metabolites and gene expression between various sites. Mussels were deployed in cages for two weeks at four sites along the Maumee River Basin, Ohio, USA. Water samples were analyzed for the presence of 220 contaminants. Hemolymph samples were collected for metabolomics and analyzed using mass spectrometry. Contaminants that significantly covaried with metabolites were identified using partial least-squares (PLS) regression. Tissue samples were collected for transcriptomics, RNA was sequenced using an Illumina HiSeq 2500, and differential expression analysis was performed on assembled transcripts. Of the 220 targeted contaminants, 69 were detected in at least one water sample. Of the 186 metabolites detected in mussel hemolymph, 43 showed significant differences between the four sites. The PLS model identified 44 contaminants that significantly covaried with changes in metabolites. A total of 296 transcripts were differentially expressed between two or more sites, 107 received BLAST hits, and 52 were annotated and assigned to one or more Gene Ontology domains. Our analyses reveal the contaminants that significantly covaried with changes in metabolites and are most likely to negatively impact freshwater mussel health and contribute to ongoing population declines in this group of highly endangered animals. Our integration of “omics” technologies provides a broad and in-depth assessment of the short-term effects of contaminants on organismal physiology. Our findings highlight which contaminants are most likely to be causing these changes and should be prioritized for more extensive toxicological testing.

## Introduction

Contaminants of emerging concern (CECs) represent a complex category of pesticides, hormones, pharmaceuticals, and personal care products that are ubiquitous in aquatic ecosystems and are increasing at a rate high enough to be considered a serious agent of global change [1–3]. These contaminants are termed “emerging” because their presence in ecosystems has only recently been discovered and information about their toxicity or behavior in the environment remains scarce [4]. The traditional approach to assessing potential health effects to humans and wildlife relies on single-contaminant toxicological testing to identify concentrations that elicit a biological response. However, this approach cannot keep pace with the growing number of CECs being detected in our water bodies and cannot predict the collective impact of complex contaminant mixtures that are naturally found in ecosystems [5]. Consequently, we have very little understanding of the potential hazards posed by most CECs and how to best prioritize contaminants for monitoring, toxicological testing, and further study.

Of all aquatic wildlife, freshwater mussels (Unionidae) might be predicted to be among the most vulnerable to CECs. These animals are efficient filter feeders, filtering up to 1 L/hr/individual [6], and purify the water by removing bacteria, algae, nutrients, metals, and a wide range of other contaminants, including CECs [7,8]. Because freshwater mussels are largely sessile animals and have a high bioaccumulation capacity [9,10], they have become great indicators of water quality and are often used in environmental monitoring and ecotoxicological studies. Unfortunately, freshwater mussel populations have experienced dramatic declines over the last 200 years, presumably due to anthropogenic activities, such as water pollution, habitat destruction or alteration, and introduction of invasive species [11]. Two-thirds of the 300 North American species are now classified as endangered, threatened, or vulnerable, and 10% have become extinct [12].

Decreasing mussel population trends have emphasized the need to advance freshwater mussel health assessment techniques [13]. “Omics” techniques, such as metabolomics and transcriptomics, have recently been shown to be effective tools for understanding freshwater mussel physiological responses to various environmental stressors [14–16] and have been used to understand effects of contaminant exposure on various other aquatic organisms [17–19]. Davis et al. [20] demonstrated the ability to use partial least-squares regression to compare aquatic CEC concentrations with metabolomic data in fathead minnows (*Pimephales promelas*), thereby showing how metabolomics can be used to shorten the list of contaminants to those that are most biologically relevant. To better understand the effects of CECs on freshwater mussels and other aquatic wildlife, we need to prioritize contaminants that are likely to elicit the greatest biological response and to better understand the nature of that response.

The objectives of this study were to (1) identify a sub-category of CECs that are most likely to adversely affect the freshwater mussel *Pyganodon grandis* (Giant Floater) and to (2) investigate differences in metabolite concentrations and gene expression between mussels deployed at variously impacted sites along the Maumee River Basin, Ohio, USA. The Maumee River watershed is mostly composed of agricultural land and is the major contributor of nutrients into Lake Erie that fuel eutrophication and associated cyanobacterial blooms [21]. The lower reaches of the watershed, near the Maumee River Bay, are further impacted by urban and suburban land use [22]. The resulting CEC mixtures from agricultural and urban land use are negatively affecting aquatic wildlife in this watershed [23–25]. Our study provides a broad assessment of the effects of CEC mixtures on freshwater mussel physiology, including recommendations for which CECs should be prioritized for further study and which metabolites and genes can function as potential biomarkers for CEC exposure.

## Methods

### Experimental design and sample collection

**Fig 1:**
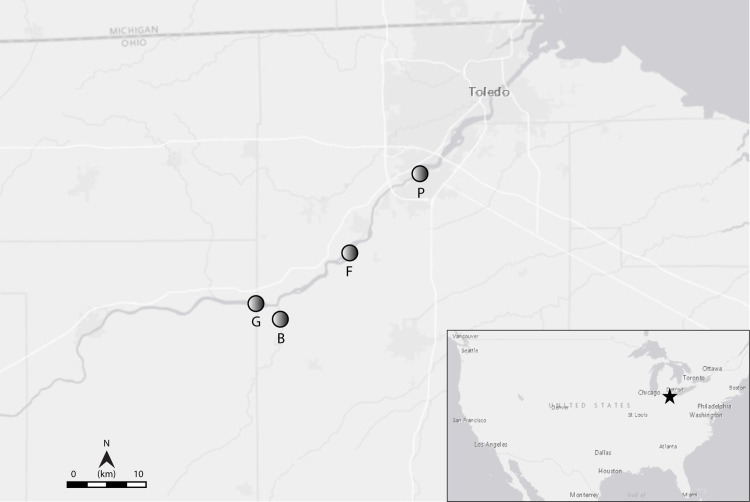
Map of Ohio showing study sites along the Maumee River. (P) Perrysburg, (F) Farnsworth Metro Park, (B) Beaver Creek, and (G) Grand Rapids Marina. The figure was produced using the USGS National Map Viewer.

Thirty-two adult *Pyganodon grandis* (giant floater) were collected from a private pond in Marion County, OH, USA on 19 May 2016. Mussels were collected under the U.S. Fish and Wildlife Service (FWS) Native Endangered Species Recovery permit TE088720-10 and access to the private pond was granted by the landowners. Mussels were transported in coolers filled with water from the collection site to the Columbus Zoo and Aquarium Freshwater Mussel Conservation and Research Center (FMCRC) near Shawnee Hills, Delaware County, OH for temporary housing. At the FMCRC, mussels were housed in tanks supplied with stream-side flow-through water from the Scioto River. On 31 May 2016, mussels were deployed in cages at four sites along the Maumee River (Fig 1). Site P was located near the outfall of the Perrysburg, OH wastewater treatment plant. Site F was located in Farnsworth Metropark, a wooded area approximately 3 miles upstream of Waterville, OH and influenced predominantly by agriculture and some septic discharge from houses along the river. Site B was located in Beaver Creek, a tributary to the Maumee River, and influenced predominantly by tilled row-crop fields and one chicken rearing concentrated animal feedlot operation (CAFO). Site G was located upstream of Independence Dam in Grand Rapids, OH and integrates a long stretch of river that is almost exclusively impacted by row-crop agriculture (and some CAFOs). One cage was placed on the substrate at each site with 8 mussels per cage. Cages were custom designed by Wanner Metal Worx, Inc. with galvanized steel bars for sides and a solid bottom and measured approximately 52 cm wide, 57 cm long, and 45 cm high.

Hemolymph samples were collected for metabolomics from each mussel on 14 June 2016. Approximately 200 μL of hemolymph were drawn from each individual by gently prying open the shell and penetrating the adductor muscle with a 25 G hypodermic needle [26]. Gill tissue samples (13–30 mg) were collected for transcriptomics from four mussels per site. Each of the hemolymph and tissue samples were transferred to a 2-mL RNase-free cryotube, snap-frozen in liquid nitrogen, and stored at -80˚C. Surface water samples were collected from each site on May 26–27, May 31-June 1, June 6–7, June 9–10, June 14–15, and June 16–17 for chemical analysis. Bottles were first rinsed with site water. Two liters of water were collected in High Density Polyethylene (HDPE) bottles and three liters were collected in baked amber glass bottles. Samples were put on ice and shipped overnight to SGS AXYS Analytical Services (Sidney, British Columbia) (AXYS) for chemical analysis.

### Metabolomics

Hemolymph samples were shipped on dry ice to Metabolon, Inc. (Durham, NC, USA), where further sample preparation and nontargeted metabolomic analyses were performed. Protein extraction was carried out using the MicroLab STAR system (Hamilton Company). Methanol was added to each sample, which was shaken vigorously for 2 min (Glen Mills GenoGrinder 2000) and then centrifuged. Extracts were split into aliquots for analysis by two separate reverse phase UPLC-MS/MS methods with positive ion mode electrospray ionization (ESI), reverse phase UPLC-MS/MS with negative ion mode ESI, and HILIC/UPLC-MS/MS with negative ion mode ESI. Organic solvents used in the extractions were removed by briefly placing samples on a TurboVap (Zymark).

Several controls were analyzed along with the experimental samples. A pooled matrix sample consisting of small volumes of each experimental sample or a pool of extensively characterized human plasma served as a technical replicate. Extracted water samples served as process blanks and a cocktail of quality control compounds were spiked into every analyzed sample. Instrument variability and overall process variability was determined by calculating the median relative standard deviation for the standards and endogenous metabolites, respectively.

Metabolites were detected using a Waters ACQUITY UPLC and a Thermo Scientific Q-Exactive high resolution/accurate mass spectrometer interfaced with a heated electrospray ionization source and Orbitrap mass analyzer. Sample extracts were dried and reconstituted in solvents compatible to each of the four detection methods. Two of the aliquots were analyzed using acidic positive ion conditions. For detection of hydrophilic compounds, the extract was gradient eluted with water and methanol (containing 0.05% perfluoropentanoic acid and 0.1% formic acid), and for detection of hydrophobic compounds, the extract was gradient eluted with methanol, acetonitrile, water, 0.05% perfluoropentanoic acid, and 0.01% formic acid. In both methods, extracts were eluted from a C18 column (Waters UPLC BEH C18-2.1x100 mm, 1.7 μm). The third aliquot was analyzed using basic negative ion conditions and extracts were gradient eluted from a separate C18 column using methanol and water with 6.5 mM ammonium bicarbonate at pH 8. The fourth aliquot was analyzed via negative ionization and extracts were eluted from a hydrophilic interaction liquid chromatography column (Waters UPLC BEH Amide 2.1x150 mm, 1.7 μm) using water and acetonitrile with 10 mM ammonium formate at pH 10.8.

Data extraction and analysis were completed using the Metabolon Laboratory Information Management System. Compound identification was obtained by comparison with more than 3,300 commercially available standards. These identifications were based on a narrow retention time/index window, mass to charge ratio (accurate mass match to the library +/- 10 ppm), and chromatographic data (MS/MS forward and reverse scores between experimental data and standards). Data were normalized to correct day-to-day instrument variation; the median of each compound was assigned a value of 1. Metabolites that differed significantly between experimental groups were identified using one-way analysis of variance (ANOVA), followed by Tukey’s honestly significant difference test.

### Transcriptomics

Tissue samples were mechanically disrupted and homogenized using a Mini-BeadBeater-8 (BioSpec Products Inc., Bartlesville, OK, USA). RNA was extracted using a RNeasy Mini Kit (Qiagen, Valencia, CA, USA). RNA concentration and integrity were measured using an Agilent 2100 Bioanalyzer (Agilent Technologies, Santa Clara, CA, USA) at The Ohio State University Comprehensive Cancer Center (Columbus, OH, USA). All samples had an RNA Integrity Number of >7.3. RNA-Seq library preparation and sequencing were performed by the Molecular and Cellular Imaging Center at the Ohio Agricultural Research and Development Center (Wooster, OH, USA). RNA-Seq libraries were prepared using the Illumina TruSeq Stranded mRNA Library Prep Kit (Illumina, Inc., San Diego, CA, USA). Libraries were sequenced on the Illumina HiSeq 2500 Sequencer (Illumina, Inc., San Diego, CA, USA) with output as 100 base pair (bp) paired-end reads.

Quality of sequencing data was assessed with FastQC (version 0.11.5; http://www.bioinformatics.babraham.ac.uk/projects/fastqc/). Quality and adapter trimming was performed using the BBMap package BBDuk (https://sourceforge.net/projects/bbmap/) (with options ktrim = r, k = 23, mink = 11, tpe, tbo, qtrim = rl. trimq = 15, maq = 20, minlen = 70). Only reads with an average Phred quality score of 20 and a minimum length of 70 bp were used in downstream analyses. *De novo* assembly of trimmed reads was performed using Trinity (version 2.6.6) [27]. using a maximum read coverage of 200. The assembly was filtered using TransRate (version 1.0.3) [28] and redundant transcripts (with a minimum similarity of 95%) were removed using cd-hit-est (version 4.7) [29]. To assess the quality of the final transcriptome assembly, the percentage of raw reads represented in the assembly was estimated by mapping with Bowtie2 (version 2.3.4.1) [30] and assembly completeness according to conserved metazoan ortholog content was assessed using BUSCO (Benchmarking Universal Single-Copy Orthologs; version 3.0.1) [31].

Transcripts were used as BLASTx (version 2.10.0) queries against the National Center for Biotechnology Information nonredundant database (downloaded April 2020) with an Expect value (E-value) cutoff of 1e^-5^ (the number of matches expected to occur by chance alone), and a hit threshold number of 20 (maximum number of matches). Functional annotation of transcripts using Gene Ontology (GO) terms and InterProScan was performed using OmicsBox (version 2.0.29) [32,33] using default parameters.

Transcript abundance was determined using Salmon (version 0.9.1) [34] and differential expression analysis was performed using the Bioconductor software package edgeR (version 3.16.5) [35]. Differentially expressed transcripts were defined as those with a False Discovery Rate (FDR) value of q<0.001 and a minimum fold-change of 2.

### Contaminant analysis

Water samples were collected by FWS to assess impacts from agriculture and urban land uses and sent to a FWS Analytical Control Facility (ACF) contracted laboratory, SGS AXYS Analytical (Sidney, BC, Canada), where they were analyzed for a total of 220 contaminants. The analyte list and methods from SGS AXYS Method MLA-035 were used to target 62 multi-residue pesticides using high resolution gas chromatography/high resolution mass spectrometry (HRGC/MS) according to the protocols described in EPA Method 1699. SGS AXYS Method MLA-075 included an analyte list of 14 hormones and 144 pharmaceuticals and personal care products (PPCPs) and was modified from the protocols described in EPA Method 1694 for the purposes of including an extended analyte list. Method MLA-075 consists of using high performance liquid chromatograph reversed phase C18 or HILIC column, coupled to a triple quadrupole mass spectrometer (LC-MS/MS).

Extraction, instrumental analysis, and analyte quantification procedures followed were in accordance with the stated SGS AXYS Methods and ACF established quality assurance and control procedures were followed. The acceptable precision of this data set was assessed utilizing reported relative percent difference values of lab generated spiked duplicates, and lab generated spikes and spiked duplicates were the principal measures of accuracy and used to assess the quality of the data.

Contaminant concentrations were considered detected if their values were equal to or above their detection limits. For contaminant concentrations (ppm) below detection limit, concentration values were set to zero. All concentration values were aggregated as a maximum value of the wet weight results. Similarly, water temperature data was aggregated as a maximum temperature value by site.

### Statistical analysis comparing water contaminants and mussel metabolites

PLS regression was used to identify water contaminants that significantly covaried with metabolites (SIMCA-13.0; Umetrics). While ordinary least squares regression is standard fare for identifying relationships between multiple explanatory variables and a single response and principal components regression is a common regression technique when the number of variables of interest exceeds the number of sampling units, neither method accommodates the covariance structure of the multiple response setting. In the case of PLS regression, the covariance structure of multiple responses is accounted for in the computations. PLS regression is shown to be an effective approach for identifying simultaneous relationships between multiple explanatory variables and multiple response variables, even in situations when the number of variables of interest exceeds the number of sampling units. PLS regression is also an effective modeling approach when there is expected to be a high degree of correlations among the explanatory variables [20].

Before utilizing PLS regression, we evaluated the extent to which metabolites in mussels differed across the four sites using one-way analysis of variance (ANOVA). It was determined that out of 186 metabolites, 143 were not significantly different across sites (p < 0.05). PLS regression was then used to assess whether these differences were related to contaminant concentrations in water samples collected at these sites. Metabolite measurements were defined as explanatory variables, while maximum contaminant concentrations and water temperature measurements were defined as response variables.

The initial model included water temperature, 69 contaminants, and 43 metabolites. The model selection routine, based on the analysis of variance testing of cross-validated predictive residuals (CV-ANOVA), removed 25 off 69 contaminants over three iterations. At each iteration, contaminants with CV-ANOVA P-values > 0.05 were excluded. The Q^2^Y measure was used to assess how well the model explained variation in individual contaminants. The Q^2^Cum measure was used to assess the overall fit of the model. As a final step, all excluded contaminants were re-introduced one-by-one back into the model, and they were retained in the model if the CV-ANOVA P-value < 0.05 and its inclusion increased Q^2^Cum measure. The final model included water temperature and 44 contaminants. Statistical analyses were conducted using R statistical software (R Core Team, 2020) and SIMCA.

## Results

### Metabolomics

In total, 186 metabolites of known identity were detected in the hemolymph of freshwater mussels. Of these, 43 showed significant differences between the four Maumee sites according to one-way ANOVA (Table 1). Most of these metabolites were involved in amino acid and lipid metabolism. Other represented pathways included cofactors and vitamins, nucleotide, xenobiotics, carbohydrate, and energy metabolism. This subset of 43 metabolites was used in downstream analyses to compare how endogenous metabolites in mussels covary with contaminant concentrations in river water.

**Table 1:** Metabolites in *Pyganodon grandis* that were significantly different (p < 0.05) between Maumee River sites according to one-way ANOVA.

Metabolite	Super pathway	Sub pathway	P	F	B	G
Betaine	Amino acid	Glycine, serine, & threonine metabolism		a		a
Dimethylglycine	Amino acid	Glycine, serine, & threonine metabolism	a	b		ab
N-acetylthreonine	Amino acid	Glycine, serine, & threonine metabolism	a	b	ab	
1-methylimidazoleacetate	Amino acid	Histidine metabolism	a	b		ab
3-methylhistidine	Amino acid	Histidine metabolism	a		a	
4-imidazoleacetate	Amino acid	Histidine metabolism	a	b	c	abc
Lysine	Amino acid	Lysine metabolism			a	a
C-glycosyltryptophan	Amino acid	Tryptophan metabolism			a	a
3-hydroxyisobutyrate	Amino acid	Leucine, Isoleucine, & valine metabolism	a	b	ab	
Isobutyrylcarnitine (C4)	Amino acid	Leucine, isoleucine, & valine metabolism			a	a
S-methylmethionine	Amino acid	Methionine, cysteine, SAM, & taurine metabolism			a	a
Trans-4-hydroxyproline	Amino acid	Urea cycle; arginine & proline metabolism	ab		a	b
N-acetylproline	Amino acid	Urea cycle; arginine & proline metabolism	a	b	ab	
Guanidinoacetate	Amino acid	Creatine metabolism	a		a	
1,3-diaminopropane	Amino acid	Polyamine metabolism	a	b	c	abc
N-acetylputrescine	Amino acid	Polyamine metabolism		a	a	
1-methylguanidine	Amino acid	Guanidino & acetamido metabolism				
4-guanidinobutanoate	Amino acid	Guanidino & acetamido metabolism	a	b	abc	c
5-oxoproline	Amino acid	Glutathione metabolism			a	a
Arabitol/xylitol	Carbohydrate	Pentose metabolism	a		ab	b
Glucuronate	Carbohydrate	Aminosugar metabolism	a	b	ab	
Succinylcarnitine (C4-DC)	Energy	TCA cycle	a		ab	b
Acetylcarnitine (C2)	Lipid	Fatty acid metabolism (acyl carnitine)	a		b	ab
3-hydroxybutyrate (BHBA)	Lipid	Ketone bodies	a		a	
Trimethylamine N-oxide	Lipid	Phospholipid metabolism	a	ab	b	
1-palmitoyl-2-alpha-linolenoyl-GPC	Lipid	Phosphatidylcholine	a		a	
1-palmitoyl-2-linolenoyl-GPC	Lipid	Phosphatidylcholine	a		a	
1-palmitoyl-2-oleoyl-GPC	Lipid	Phosphatidylcholine	ab		a	b
1-palmitoyl-2-palmitoleoyl-GPC	Lipid	Phosphatidylcholine	ab		a	b
1,2-dilinoleoyl-GPC	Lipid	Phosphatidylcholine	a		a	
Glycerophosphoglycerol	Lipid	Glycerolipid metabolism	a			a
N6-carbamoylthreonyladenosine	Nucleotide	Purine metabolism, adenine containing	a		a	
N2,N2-dimethylguanine	Nucleotide	Purine metabolism, guanine containing	a		ab	b
3-ureidopropionate	Nucleotide	Pyrimidine metabolism, uracil containing			a	a
2’-O-methylcytidine	Nucleotide	Pyrimidine metabolism, cytidine containing			a	a
Nicotinamide riboside	Cofactors & vitamins	Nicotinate & nicotinamide metabolism		a	ab	b
Trigonelline (N’-methylnicotinate)	Cofactors & vitamins	Nicotinate & nicotinamide metabolism	a		a	
Pterin	Cofactors & vitamins	Pterin metabolism	a		a	
Pyridoxal	Cofactors & vitamins	Vitamin B6 metabolism	a	b	ab	
Pyridoxate	Cofactors & vitamins	Vitamin B6 metabolism	a		a	
P-cresol sulfate	Xenobiotics	Benzoate metabolism		a	a	
Stachydrine	Xenobiotics	Food component/plant	a	b		ab
Salicylate	Xenobiotics	Drug		a	ab	b

Pairs of same lowercase letters indicate significant differences between sites according to Tukey’s test. P = Perrysburg. F = Farnsworth Metropark. B = Beaver Creek. G = Grand Rapids.

### Transcriptomics

Illumina sequencing produced 192,694,999 raw reads. The final transcriptome assembly consisted of 200,761 transcripts with a mean length of 714 bp, N50 of 1,104 bp (50% of transcripts are equal to or larger than this value), and guanine-cytosine (GC) content of 34.93% (Table 2). Bowtie2 mapped 93.63% of trimmed reads to the transcriptome assembly. BUSCO analysis indicated that the assembly produced 824 (84.3%) complete, 103 (10.5%) fragmented, and 51 (5.2%) missing BUSCOs. Data were archived in GenBank under BioProject accession number PRJNA885125 (https://www.ncbi.nlm.nih.gov/bioproject/).

**Table 2:** Summary statistics for sequencing and transcriptome assembly.

Statistic	Value
Raw reads produced by Illumina sequencing	192,694,999
Estimate of reads used in final assembly	93.63%
Total assembled transcripts	200,761
Total assembled bases	143,362,661
Mean transcript length	714 bp
Median transcript length	412 bp
N50	1,104 bp
GC content	34.93%

**Fig 2:**
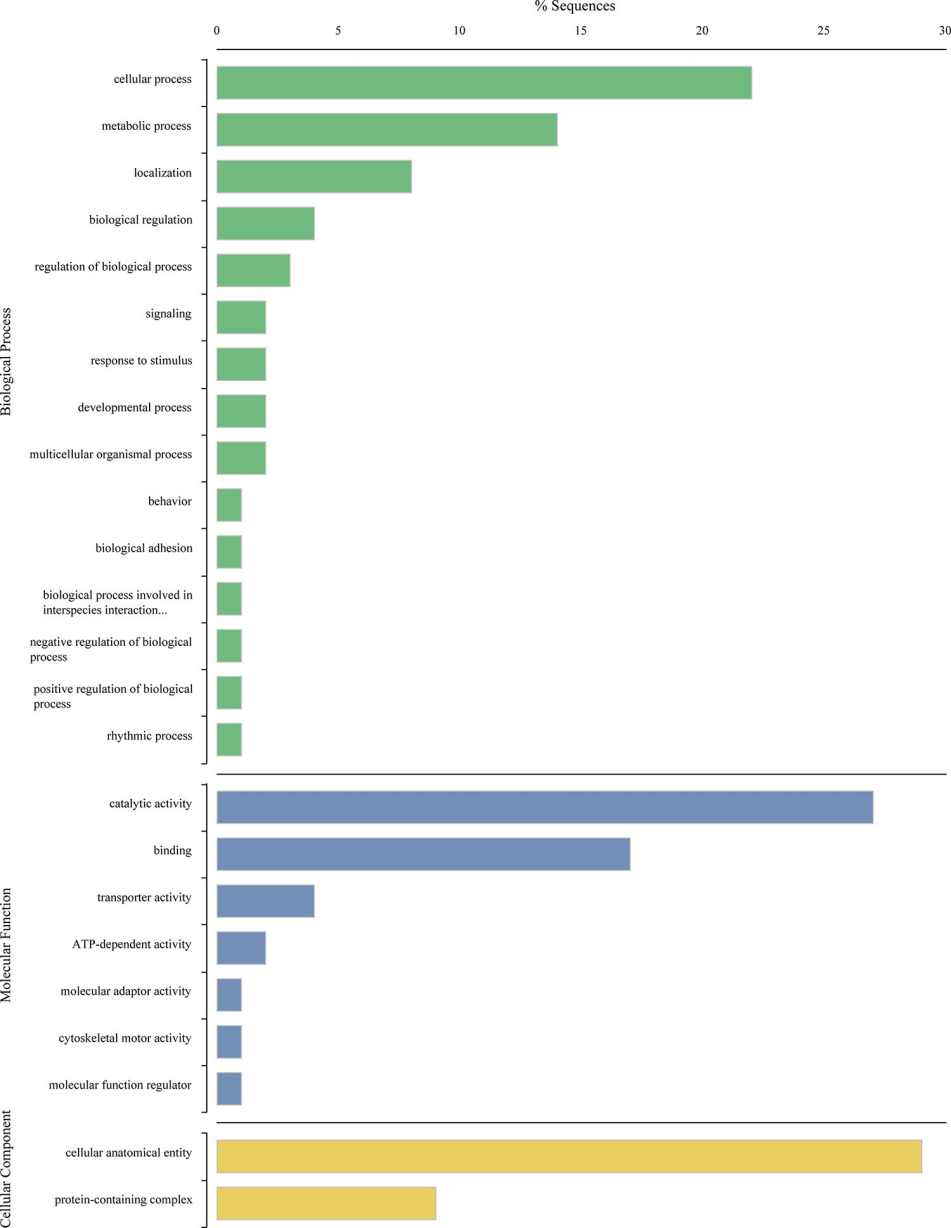
Gene Ontology terms and the percentage of corresponding differentially expressed transcripts between sampling sites.

Of the 200,761 transcripts assembled by Trinity, 49,760 (24.79%) received BLAST hits, and 27,095 of these were annotated with GO terms. A total of 296 transcripts were differentially expressed between two or more Maumee sites (S1-S7 Tables in S1 File). Of these transcripts, 107 received BLAST hits and 52 were annotated. Transcripts were assigned to one or more of the GO domains: “biological process” (24 transcripts), “molecular function” (39 transcripts), and “cellular component” (29 transcripts). The most common second-level categories within biological process were “cellular process”, “metabolic process”, and “localization” (Fig 2). The most common categories within molecular function were “catalytic activity” and “binding”, and those in cellular component included “cellular anatomical entity” and “protein-containing complex” (Fig 2).

### Contaminants of emerging concern

Of the 220 targeted contaminants, 69 were detected in at least one water sample. Detected contaminants included 10 hormones, 21 pesticides, and 38 PPCPs (Table 3). Atrazine, caffeine, DEET, desethylatrazine, iopamidol, metformin, and metribuzin were among the top ten contaminants detected at all sites (Table 4). Other top ten contaminants that were detected in at least one site included androsterone, fluoxetine, simazine, sulfamethoxazole, and valsartan.

**Table 3:** Detected contaminants of emerging concern in water samples.

Contaminant class	Number sampled	Number detected
Hormones	14	10
Pesticides	62	21
Pharmaceuticals and personal care products	144	38

**Table 4:** Top ten contaminants of emerging concern detected in water at the sampling sites.

Site	Contaminant	Class	Concentration (ppm)	# of observations
Perrysburg (P)	Iopamidol	PPCP	0.0007610	6
	Atrazine	Pesticide	0.0007205	6
	Metformin	Hormone	0.0003890	6
	Metribuzin	Pesticide	0.0003495	6
	DEET	PPCP	0.0000926	6
	Desethylatrazine	Pesticide	0.0000804	6
	Simazine	Pesticide	0.0000540	6
	Caffeine	PPCP	0.0000538	6
	Sulfamethoxazole	PPCP	0.0000412	6
	Valsartan	PPCP	0.0000247	6
Farnsworth (F)	Iopamidol	PPCP	0.0007165	6
	Atrazine	Pesticide	0.0007065	6
	Metribuzin	Pesticide	0.0005375	6
	Desethylatrazine	Pesticide	0.0004047	6
	Metformin	Hormone	0.0003175	6
	Simazine	Pesticide	0.0001302	6
	Caffeine	PPCP	0.0000602	6
	DEET	PPCP	0.0000512	6
	Androsterone	PPCP	0.0000465	6
	Fluoxetine	PPCP	0.0000335	6
Beaver Creek (B)	Atrazine	Pesticde	0.0010800	6
	Metformin	Hormone	0.0004485	6
	Iopamidol	PPCP	0.0002055	6
	Metribuzin	Pesticide	0.0001760	6
	Desethylatrazine	Pesticide	0.0000614	6
	DEET	PPCP	0.0000517	6
	Androsterone	PPCP	0.0000397	6
	Caffeine	PPCP	0.0000306	6
	Fluoxetine	PPCP	0.0000276	6
	Sulfamethoxazole	PPCP	0.0000219	6
Grand Rapids (G)	Iopamidol	PPCP	0.0007920	6
	Atrazine	Pesticide	0.0006730	6
	Metribuzin	Pesticide	0.0003640	6
	Desethylatrazine	Pesticide	0.0002948	6
	Metformin	Hormone	0.0002855	6
	Simazine	Pesticide	0.0001199	6
	DEET	PPCP	0.0000696	6
	Caffeine	PPCP	0.0000659	6
	Sulfamethoxazole	PPCP	0.0000240	6
	Fluoxetine	PPCP	0.0000235	6

### Comparison of water contaminants and mussel metabolites

Of the 220 contaminants analyzed, 151 were not detected at any site and were dropped from the analysis. Using PLS regression, a global model was fit which included water temperature, 69 contaminants, and 43 metabolites. Using CV-ANOVA values, 25 out of 69 contaminants were removed over the course of three iterations.

The final PLS model had three principal components determined such that the covariance between the principal component scores for the explanatory variables and principal component scores for the response variables was maximized. These principal components explained 44.3%, 13.4%, and 6.1% of the variance in explanatory variables, and 14.2%, 29.1%, and 20.8% of the variance in the response variables, respectively. The final model identified 44 contaminants that significantly covaried with changes in metabolites in mussels (Table 5). The overall predictive power of the model, as measured by the cumulative Q^2^, was 0.495 (vs. 0.442 for the global model). The strength of the relationship between a contaminant and metabolites, as measured by Q^2^Y value, ranged from 0.378 to 0.647. The five contaminants that had the strongest relationship with metabolites were endosulfan sulfate (0.647), gamma chlordane (0.634), alpha chlordane (0.632), moxifloxacin (0.632), and alprazolam (0.627).

**Table 5:** Contaminants that significantly covaried with changes in metabolites in mussels, as indicated by the partial least-squares model.

Contaminant	Q^2^Y	CV-ANOVA
Endosulfan sulfate	0.647	0.000120
Gamma chlordane	0.634	0.000180
Alpha chlordane	0.632	0.000188
Moxifloxacin	0.632	0.000188
Alprazolam	0.627	0.000221
Endrin	0.620	0.000237
Heptachlor epoxide	0.590	0.000529
DEET	0.576	0.000908
Aldrin	0.564	0.001033
Sulfamethazine	0.562	0.000732
Atrazine	0.550	0.002526
Metformin	0.543	0.002472
Ametryn	0.536	0.002474
Amitriptyline	0.532	0.004767
Sertraline	0.532	0.002299
Metribuzin	0.520	0.003056
Dieldrin	0.513	0.004149
Amsacrine	0.512	0.002648
Prednisone	0.512	0.002648
Hcb	0.509	0.004929
Amthetamine	0.505	0.003155
Androstenedione	0.503	0.005313
Chlorothalonil	0.501	0.003484
Desethylatrazine	0.465	0.008531
Sulfadimethoxine	0.460	0.010555
Lincomycin	0.446	0.015580
Chlorpyriphos methyl	0.417	0.023789
Endrin ketone	0.417	0.023789
Diphenhydramine	0.398	0.037235
10_hydroxy_amitriptyline	0.397	0.032633
Albuterol	0.397	0.032633
Azithromycin	0.397	0.032633
Cimetidine	0.397	0.032633
Citalopram	0.397	0.032633
Clarithromycin	0.397	0.032633
Cocaine	0.397	0.032633
Codeine	0.397	0.032633
Cyanazine	0.397	0.032633
Desmethyldiltiazem	0.397	0.032633
Metoprololfl	0.397	0.032633
Ofloxacin	0.397	0.032633
Oxycodone	0.397	0.032633
Verapamil	0.397	0.032633
Atenolol	0.378	0.042075

Metabolites that were significantly related to changes in contaminant concentrations and temperature were determined using variable importance on the projection (VIP) values ≥ 1.0 from the final PLS model. VIP values summarize the overall importance of each x variable (i.e., metabolite) on all y variables (i.e., contaminant concentrations and temperature). Similar to Davis et al. [20], metabolites with VIP values statistically greater than 1.0 (i.e., p < 0.05) were considered to be the most relevant for explaining variation in contaminant concentration levels. Of the metabolites with VIP values greater than 1.0, 4-imidazoleacetate, stachydrine, salicylate, and betaine loaded positively on the first component of the PLS model, whereas metabolites 3-hydroxybutyrate (BHBA), 3-ureidopropionate, acetylcarnitine (C2), glycerophosphoglycerol, trans-4-hydroxyproline, P-cresol sulfate, and trimethylamine N-oxide loaded negatively on this component. For the second component of the PLS model, metabolites 3-hydroxybutyrate (BHBA), glycerophosphoglycerol, trans-4-hydroxyproline, and salicylate loaded positively and 3-ureidopropionate, 4-imidazoleacetate, acetylcarnitine (C2), betaine, P-cresol sulfate, stachydrine, and trimethylamine N-oxide loaded negatively.

## Discussion

### Contaminants strongly correlated with metabolic changes

The quickly increasing occurrence and detection of CECs requires scientists and managers to prioritize contaminants for monitoring and toxicological testing. Those contaminants that elicit the greatest biological response are more likely to have detrimental effects on organismal health. By narrowing down the list of contaminants detected at the Maumee River sites to those that significantly covary with relative changes in mussel metabolite levels, we have effectively prioritized the most important contaminants for further study (Table 5). Furthermore, this sub-category of contaminants is ordered by the strength of correlation between a contaminant and metabolites, as measured by the Q^2^Y value.

Our study showed that, of all contaminants present in the Maumee River, endosulfan sulfate had the strongest correlation with metabolic changes in *P*. *grandis* (Table 5). Endosulfan sulfate is a breakdown product of endosulfan, an organochlorine insecticide that has been banned or phased out in multiple countries due to its environmental persistence, bioaccumulation, and high toxicity [36]. Endosulfan exposure has been linked with metabolic alterations in many aquatic organisms, including fish [37], amphibians [38], and invertebrates [39]. Although endosulfan is banned in the United States, its high environmental persistence and long-range atmospheric transport [40] are likely responsible for our ability to detect its breakdown product in the Maumee River.

Chlordane is another persistent organochlorine insecticide that was used in the United States on agricultural crops, lawns, and gardens, and as termite control until it was banned in 1988 [41,42]. Chlordane consists of over 140 components, including the major constituents alpha and gamma chlordane, also known as *cis*- and *trans*-chlordane, respectively [43], which showed the second and third strongest correlations with metabolic changes in our mussels (Table 5). Although chlordane has low solubility in water, it adheres to soil particles and dissolved organic carbon, which can then be transported into aquatic systems through runoff from urban and agricultural land [44] and taken up by a wide range of aquatic organisms, including invertebrates, fish, birds, and mammals [41,45].

Unlike the top three contaminants exhibiting the strongest correlations with metabolic changes in mussels, which are all components of banned persistent organochlorine insecticides, moxifloxacin (Table 5) is an essential quinolone antibiotic used to treat common infections such as conjunctivitis, pneumonia, and sinusitis by inhibiting bacterial DNA replication [46,47]. Gilroy et al. [48] suggested that moxifloxacin posed low risk to freshwater mussels since laboratory exposure at 0.01, 0.1, 1, 10, and 100 mg/L did not affect survival, oxygen consumption, hemocyte density or glutathione S-transferase activity in *Lampsilis siliquoidea* and only affected algal clearance rate and larval viability at concentrations exceeding expected environmental concentrations. However, our results show that moxifloxacin does significantly affect freshwater mussels at environmentally relevant concentrations and is among the top five contaminants in the Maumee River with strongest correlations to metabolic changes.

Alprazolam (sold under the brand name Xanax) has the fifth strongest correlation to metabolic changes in mussels (Table 5). This benzodiazepine has been widely used in medicine since the 1960s to treat generalized anxiety, panic attacks, and depression and is one of the most prescribed psychoactive drugs [49]. Aplrazolam has a long half-life in aquatic systems [50] and is not well removed through conventional wastewater treatment systems [51]. Studies have looked at the effects of exposure in freshwater mussels to psychoactive drugs in the selective serotonin reuptake inhibitor class, such as fluoxetine and sertraline [52–55], but little attention has been paid to aplrazolam. However, our results show that alprazolam has a stronger correlation with metabolic changes in mussels than sertraline, which has the 15^th^ strongest correlation (Table 5), and fluoxetine, which is not included in this subset of contaminants despite being found in higher concentrations in Maumee River water than most other contaminants (Table 4). We suggest that alprazolam, along with other contaminants in Table 5 that show strong correlations with metabolic changes in mussels, be prioritized for ecotoxicological testing in freshwater mussels.

Although the subset of contaminants that strongly correlate with metabolite changes in *P*. *grandis* (Table 5) are likely negatively impacting freshwater mussels and should be prioritized for toxicological testing, this does not mean that the contaminants excluded from this list should be ignored. It is possible that a contaminant with a strong biological effect may have been excluded if its concentration is similar between the four Maumee River sites. In this case, any induced metabolic changes in mussels may also be similar across sites and not detected in our study. Nevertheless, our analysis provides an initial screening process useful for deciding which contaminants to prioritize for further study.

### Potential metabolite biomarkers

Of the 43 metabolites that showed significant differences between the four Maumee sites, 11 were significantly related to changes in contaminant concentrations and temperature. Most of these metabolites are part of pathways involved in lipid metabolism: acetylcarnitine (C2) (fatty acid metabolism), 3-hydroxybutyrate (ketone bodies), trimethylamine N-oxide (phospholipid metabolism), and glycerophosphoglycerol (glycerolipid metabolism). Changes in lipids and their metabolites have been observed in other bivalves such as *Mytilus galloprovincialis* in response to wastewater treatment plant effluent [56] and in *Crassostrea hongkongensis* in response to copper exposure [57]. In general, a wide range of pollutants, from organic pollutants to heavy metals, can cause significant alterations to lipid metabolism in humans and wildlife [58]. Other metabolites that were significantly related to changes in contaminant concentrations and temperature were part of pathways involved in amino acid (betaine, 4-imidazoleacetate, and trans-4-hydroxyproline) and xenobiotic metabolism (p-cresol sulfate, stachydrine, and salicylate) and one metabolite in nucleotide metabolism (3-ureidopropionate). These 11 metabolites may serve as potential biomarkers, or biological endpoints, which respond to changes in contaminant exposure [59,60].

### Differential gene expression

Integrating transcriptomic and metabolomic techniques provides a direct link between an environmental stressor and the response of an organism. A change in the environment typically alters metabolite levels first before changes in the transcriptome or proteome can be observed [61], which is why we chose to analyze correlations between contaminant and metabolite levels directly. However, because metabolites are interconverted by enzymes that are the products of gene expression, including information about differential gene expression provides more information about an organism’s response to environmental changes at a different physiological level. Here, we discuss some of the differences in gene expression observed between mussels deployed at the various Maumee River sites.

Most transcripts assigned to the GO domain “biological process” belong to the second-level categories “cellular process” and “metabolic process” (Fig 2). These transcripts are involved in processes carried out at the cellular level and the chemical reactions and pathways, such as anabolism and catabolism, which transform chemical substances. For example, glutathione S-transferase is involved in the detoxification process by conjugating glutathione to xenobiotic substances and is a marker of oxidative stress in aquatic biota [62]. The transcript coding for glutathione S-transferase was differentially expressed between mussels at the various Maumee Sites (S1 Table in S1 File) and has previously been shown to be up-regulated in response to translocation stress [16]. Other examples of differentially expressed transcripts involved in cellular metabolic processes included those coding for cytochrome c oxidase subunit II, mitochondrial ATP synthase subunit b, and DNA-directed RNA polymerase I and III subunit.

Most transcripts assigned to the GO domain “molecular function” belong to the second-level categories “catalytic activity” and “binding” (Fig 2). These transcripts are involved in the interaction of molecules with one another and in biochemical reactions in which substrates are catalyzed by enzymes. Many of these transcripts code for proteins with oxidoreductase activity, which plays an important role in detoxification pathways in aquatic organisms [63,64]. For example, a transcript coding for cytochrome p450 was differentially expressed between our mussels (S1 Table in S1 File) and has been shown to alter expression in rainbow trout in response to the contaminants benzo[a]pyrene and ethinylestradiol [65] and in the freshwater mussel *Elliptio complanata* in response to wastewater effluent [66].

Most transcripts assigned to the GO domain “cellular component” belong to the second-level category “cellular anatomical entity” (Fig 2). The majority of these transcripts code for proteins involved in intracellular anatomical structure and the cell membrane. Examples include small nuclear ribonucleoprotein E, dynein heavy chain 3, intraflagellar transport protein 56, and multidrug resistance-associated protein (S1 Table in S1 File). Both metal and non-metal contaminants and urban sewage have been shown to degrade cellular structures and cytoskeleton proteins in other bivalves [67–69], a phenomenon observed in many other aquatic organisms [70].

## Conclusion

Due to the number of CECs found in aquatic systems, metabolomics provides a powerful screening tool for chemicals that elicit a biological response and, therefore, should be prioritized for more extensive toxicological testing [71]. Here, we used “omics” techniques and PLS regression to narrow down the list of contaminants found in the Maumee River basin from a total of 69 to those 44 that showed significant correlation with endogenous metabolites in the freshwater mussel *P*. *grandis*, resulting in a 36% reduction in CECs of interest. These 44 CECs are most likely to negatively impact freshwater mussel health and, therefore, contribute to ongoing population declines of this group of highly endangered animals. We also discussed how differences in gene expression between mussels deployed at the various sites revealed significant alterations in detoxification and oxidative stress metabolism. The integration of metabolomics, transcriptomics, and CEC analyses provides a broad and in-depth assessment of how contaminants affect organismal physiology and which contaminants are most likely to be causing these changes. Furthermore, these methods can be applied to any organism, which provides a great opportunity for comparative studies among different taxa and to any river, lake, pond, or other aquatic system. The ability to rank and prioritize the severity of different environmental stressors, not just contaminants of emerging concern, is of utmost importance in today’s world where ecosystems are faced with multiple stressors ranging from climate change and pollution.

## Supporting information

S1 File (XLSX)
